# Lifestyle habits and depressive symptoms in Norwegian adolescents: a national cross-sectional study

**DOI:** 10.1186/s12889-021-10846-1

**Published:** 2021-04-28

**Authors:** Annette Løvheim Kleppang, Siri Håvås Haugland, Anders Bakken, Tonje Holte Stea

**Affiliations:** 1 Department of Health and Nursing Science, University of Agder, Postboks 422 4604, Kristiansand, Norway; 2 Department of Psychosocial Health, University of Agder, Kristiansand, Norway; 3 NOVA-Norwegian Social Research, OsloMet-Oslo Metropolitan University, Oslo, Norway; 4 Department of Child and Adolescence Mental Health, Sørlandet Hospital, Kristiansand, Norway

**Keywords:** Adolescents, Lifestyle habits, Symptoms of depression, Norway

## Abstract

**Background:**

This study’s purpose was to examine the association between a broad range of lifestyle habits and depressive symptoms in Norwegian adolescents.

**Methods:**

This study was based on national, self-reported, cross-sectional data from the Ungdata Surveys, conducted in 2017–2019. The target group comprised 244,250 adolescents (ages 13–19). Binominal logistic regression was used to analyse the association between lifestyle habits (physical activity, social media use, gaming, dietary habits, smoking, smokeless tobacco, alcohol intoxication) and depressive symptoms. The outcome measure was defined as a high level of depressive symptoms (≥80th percentile). Separate analyses were performed for boys and girls, and all models were adjusted for perceived family economy, parental higher education and age.

**Results:**

The odds of having depressive symptoms were significantly lower among those who reported being physically active at least 3 times per week (OR; boys: 0.81, girls: 0.83), used social media ≤3 h per day (OR; boys: 0.65, girls: 0.70), engaged in gaming ≤3 h per day (OR; boys: 0.72, girls: 0.77), were non-smokers (OR; boys: 0.74, girls: 0.72) and had not experienced alcohol intoxication during the previous 12 months (OR; boys: 0.66, girls: 0.67). Furthermore, the results indicated a significant inverse association between depressive symptoms and high consumption of a range of healthy food items and low consumption of unhealthy food and beverages among girls. Similar tendencies were found among boys (OR; 0.77–0.91). Finally, higher adherence to healthy lifestyle habits was associated significantly with lower odds of having depressive symptoms among both genders (OR; boys: 0.40, girls: 0.52).

**Conclusions:**

A healthier lifestyle was associated with lower odds of having depressive symptoms. Additional research is needed to confirm a possible causal relationship.

## Background

Globally, depression is the fourth leading cause of illness and disability among adolescents ages 15–19 [1], and depressive symptoms continue to increase, especially among girls [2, 3]. Therefore, identifying factors that can reduce the risk of depressive symptoms is essential. Some studies that have examined associations between lifestyle habits and depressive symptoms among adolescents identified multifactorial associations [4–6], while others revealed gender differences [5, 7]**.** A recent prospective study reported that adolescents meeting recommendations for multiple lifestyle behaviours – including diet, physical activity, screen time and sleep – experienced fewer health care encounters due to mental illness than those meeting recommendations for a single behaviour [8]. However, evidence confirms that meeting dietary recommendations developed to improve physical health also most likely may exert a positive effect on mental health [9]. Overall, systematic reviews among children and adolescents have concluded that healthy dietary habits seem to be associated with reduced risk of depression and improved mental health, whereas unhealthy dietary habits seem to be associated with increased risk of depression and poorer mental health [10, 11]. Due to different methodological challenges and partly inconsistent results from previous studies [11], national and international health authorities have emphasised the need for increased knowledge about possible associations between dietary habits and mental health problems, including depressive symptoms [12, 13].

Furthermore, systematic reviews have concluded that decreased sedentary behaviour and increased physical activity levels are associated with reducing depression and, to a certain extent, psychological distress in children and adolescents [14, 15]. A meta-analysis of prospective cohort studies also reported that higher levels of physical activity may protect against the future development of depression, regardless of age [16]. However, a longitudinal study found no association between physical activity level (measured objectively) and depression [17].

High levels of screen time – which includes time spent on the web surfing, gaming, social media messaging and TV/movie watching – also have been associated with depressive symptomatology in adolescents [18, 19]. A recent systematic review of reviews concluded that although few studies have been conducted, there was moderate to strong evidence confirming an association between screen time and depression, but limited evidence indicated an association between social media use and symptoms of depression [20].

Finally, a strong association has been found between alcohol intoxication and prevalence of depressive symptoms among Norwegian adolescents, and that those with depressive symptoms are more likely to have experienced an earlier onset of alcohol use and more frequent alcohol consumption [21, 22]. Smoking also has been associated with symptoms of depression [23–25], and a recent longitudinal study reported that tobacco use during adolescence appears to influence the onset of depressive symptoms, especially among boys [26].

Low socioeconomic status (SES) is associated with both lifestyle habits [21, 27, 28] and mental health problems [29, 30]; therefore, it is an important confounder to consider when analysing the relationship between lifestyle habits and depressive symptoms.

In the Norwegian governmental strategy plan for good mental health, special attention has been paid to promoting good mental health among children and adolescents, emphasising health-promoting lifestyles [12]. To develop tailored measures, it is crucial to have knowledge about the range of protective and promotive factors that influence adolescents’ mental health [31]. To the best of our knowledge, few previously published studies have reported on multiple unhealthy and healthy lifestyle habits among a large sample of adolescents concerning depressive symptoms. The present study aims to examine the association between physical activity, social media use, gaming, dietary habits, substance use and depressive symptoms, adjusting for age, family economy and parental higher education in a large national sample of Norwegian adolescents.

## Methods

This study was based on data from the Ungdata Survey, an ongoing, nationally representative study of students from grades 8 to 13 across almost all municipalities in Norway. The Ungdata Survey aims to investigate adolescents’ health and well-being at the municipal and national levels (see www.ungdata.no). The survey is conducted by the Norwegian Social Research (NOVA) at Oslo Metropolitan University in a collaboration with all Regional Drug and Alcohol Competence Centres (KoRus). The surveys are financed partially by the Norwegian Directorate of Health and gauge different aspects of adolescents’ lives, i.e., health issues, local environment, school issues, diet and beverage consumption, relationships with friends and parents, leisure time activities and symptoms of depression. They also include questions about tobacco use and alcohol consumption.

In this study, data from surveys conducted from 2017 to 2019 were used. During this period, Ungdata was conducted in 417 out of 435 Norwegian municipalities. The few municipalities that did not participate were small municipalities with a very low population of adolescents. In the participating municipalities, students from almost all secondary schools participated and were not given any incentives. Parents and students were informed via mail in advance, and parents for adolescents ages 13–17 were assured that they could withdraw their children from participation at any time. Students decided in school whether they wanted to participate after being informed that participation was voluntary and that they could skip questions that they did not want to answer. The study was conducted as a web-based questionnaire administered at school during school hours with a teacher or an administrator present to answer questions. The students used approximately 30–45 min to complete the questionnaire. Altogether, 244,250 adolescents participated, for an overall participation rate of 87% in lower secondary school and 73% in upper secondary school [32]. Both genders were represented equally, as were students at different grade levels in the sample, compared with official student statistics [33]. Most of the participants (94%) answered all or almost all variables used in this paper. Independent researchers who did not participate in data collection managed and analysed an anonymous data file. The study was conducted in line with the Declaration of Helsinki, and the Norwegian Centre for Research Data (NSD) approved all privacy aspects of the study.

Table 1 provides the Ungdata Survey 2017–2019 questions, response alternatives and variable definitions included in this study.

**Table 1:** Ungdata Survey 2017–2019: questions, response alternatives and variable definitions

Questions	Response alternatives	Variable definitions
***Symptoms of depression***		
*During the past week, have you been affected by any of the following issues:*		
Felt that everything is a struggle (item 1)	Not been affected at all, not been affected much, been affected quite a lot, been affected a great deal.	High level of depressive symptoms ≥80th percentiles
Had sleep problems (item 2)		
Felt unhappy, sad or depressed (item 3)		
Felt hopelessness about the future (item 4)		Low level of depressive symptoms <80th percentiles
Felt stiff or tense (item 5)		
Worried too much about things (item 6)		
***Physical activity***		
How often do you do physical activity, which gets you out of breath or makes you sweaty?	Never, rarely, 1–2 times a month, 1–2 times a week, 3–4 times a week, at least 5 times a week.	Active participants ≥3 times a week
***Social media use***		
Think about what you do a normal day: How much time do you spend on the following things: social media (facebook, Instagram etc.)	No time, < 30 min, 30 min - 1 h, 1–2 h, 2–3 h, > 3 h	≤ 3 h per day, > 3 h per day
***Gaming on computer/TV***		
Think about what you do a normal day: How much time do you spend on the following things: gaming on computer/TV?	No time, < 30 min, 30 min - 1 h, 1–2 h, 2–3 h, > 3 h	≤ 3 h per day, > 3 h per day
***Gaming on telephone/tablets***		
Think about what you do a normal day: How much time do you spend on the following things: gaming on telephone/tablets?	No time, < 30 min, 30 min - 1 h, 1–2 h, 2–3 h, > 3 h	≤ 3 h per day, > 3 h per day
***Smoking***		
Do you smoke?	I’ve never smoked, I used to smoke, but I’ve stopped completely now, I smoke less than once a week, I smoke every week, but not every day, I smoke every day.	Current use, no current use
***Smokeless tobacco***	I’ve never used smokeless tobacco, I used to use smokeless tobacco previously but quit, I use smokeless tobacco less than once a week, I use smokeless tobacco weekly but not daily, I use smokeless tobacco daily	Current use, no current use
***Alcohol intoxication (previous 12 months)***	Never, once, 2–5 times, 6–10 times, more than 11 times	Any intoxication episode, no intoxication episodes
***Diet and beverage consumption***		
How often do you eat or drink the following**?**		
Vegetables	Never, less than once a week, once a week, 2–3 times a week, 4–6 times a week, every day, several times a day	Once a day and more, less than once day
Fruit		
Whole grain bread		
Fish		Once a week or more, less than once a week
Salty snacks		≥ 4 times a week, < 4 times a week
Candy		
Sugar-sweetened beverages		
Diet beverages		
Energy drinks		
***Gender***		
Are you a boy or a girl?	Boy, girl	
***Perceived family economy***		
Financially, has your family been well off, or badly off, over the past years?	We have been well off the whole time, we have generally been well off, we have neither been well off nor badly off, we have generally been badly off, we have been badly off the whole time	Good economy, nor bad or good economy, bad economy
***Parents higher education*** Did your father and mother go to university or to a university college? Select one answer for each parents. If you are not in touch with one or both of your parents, skip the question about that parent.	Yes, no	Both parents, One of the parents, None of the parents

### Measures

#### Symptoms of depression

The six items that measured depressive symptoms were derived from a scale based on Hopkins Symptom Checklist 90 [34, 35]. The depressive-symptom scale has been evaluated psychometrically among Norwegian adolescents and has demonstrated good reliability (Person Separation Index: 0.802). As a whole, the scale works reasonably well on a general level [36].

#### Lifestyle habits

The adolescents provided information about physical activity, screen time, tobacco use, alcohol intoxication and daily diet, i.e., consumption of food and beverages (see Table 1). The cut-off value for physical activity (≥3 times a week) is used to measure moderate to high levels of physical activity. The cut-off value for screen use [37], consumption of healthy and unhealthy beverages, and food [38, 39] has been used in similar populations.

#### Background variables

Perceived family economy and parental higher education were assessed using subjective measures (see Table 1), as in extant studies, to measure socioeconomic background [5, 40]. Class level was applied as a proxy for adolescent age.

#### Construction of the lifestyle habit score

To reflect on and quantify a broad range of lifestyle habits related to public health and health promotion, we constructed a lifestyle habit score (LSH) that assesses adherence to diet and beverage consumption (see Table 1), physical activity, screen use, gaming, smoking, smokeless tobacco and alcohol intoxication habits, along with their potentially healthy and unhealthy properties. Each of the 15 subscales was dichotomised, and the scoring values for each subscale were zero or one. The 15 lifestyle indicators (subscales) were chosen to comprise the score based on a combination of potential lifestyle factors. The LSH score was computed by adding up the 15 dichotomised subscale scores, yielding a possible scoring range of 0–15, with a higher LSH score indicating adherence to several healthy lifestyle habits (mean; 4.08, SD; 1.94). For description and analysis, the LSH score was divided into five groups based on quintiles.

#### Analysis

The data were analysed using IBM SPSS Statistics 25 software. A descriptive contingency table was created, and the study population was stratified according to depressive symptoms and gender. Baseline characteristics were presented as proportions, with 95% confidence intervals (CIs) in each stratum. Multivariate binomial logistic regression analyses were used to examine the relationship between healthy and unhealthy lifestyle habits and depressive symptoms, and between LSH scores and depressive symptoms. A *p*-value of ≤0.05 was required for statistical significance. Multivariate analyses were conducted separately for boys and girls, and were adjusted for age, perceived family economy and parents’ higher education. The results were presented as odds ratios (ORs), with 95% CIs.

## Results

Table 2 provides the baseline characteristics of the study population, ages 13–19, during the 2017–2019 period, according to depressive symptoms and gender.

**Table 2:** Ungdata Survey: Baseline characteristics (percentales and 95%CI) of adolescents aged 13–19 years in 2017–2019, according to depressive symptoms and gender

Variables	Total (*n* = 241,730)		Boys (*n* = 116,780)		Girls (*n* = 121,583)	
	Low level of depressive symptoms (*n* = 187,857)	High level of depressive symptoms (*n* = 53,873)	Low level of depressive symptoms (*n* = 101,105)	High level of depressive symptoms (*n* = 14,232)	Low level of depressive symptoms (*n* = 82,353)	High level of depressive symptoms (*n* = 38,254)
**Lifestyle habits**						
***Physical activity level***						
≥ 3 times a week	59.3 (59.1–59.5)	47.8 (47.4–48.3)	62.7 (62.4–63.0)	54.3 (53.5–55.1)	55.0 (54.7–55.4)	45.5 (45.0–46.0)
***Social media use***						
≤ 3 h per day	77.8 (77.6–77.9)	59.0 (58.6–59.4)	84.5 (84.3–84.7)	71.6 (70.9–72.4)	69.7 (69.4–70.0)	54.4 (53.9–54.9)
***Gaming***						
≤ 3 h per day	81.9 (81.7–82.0)	79.7 (79.4–80.1)	74.1 (73.8–74.3)	63.3 (62.5–64.1)	91.3 (91.1–91.5)	85.7 (85.4–86.1)
***Smoking***						
No current use	90.7 (90.6–90.9)	81.6 (81.2–81.9)	88.5 (88.3–88.7)	75.4 (74.7–76.1)	93.5 (93.3–93.7)	84.0 (83.6–84.4)
***Smokeless tobacco***						
No current use	89.7 (89.6–89.9)	80.7 (80.4–81.0)	87.8 (87.6–88.0)	76.0 (75.3–76.7)	92.3 (92.1–92.4)	82.6 (82.3–83.0)
***Alcohol intoxication (previous 12 months)***						
No intoxication episodes	71.6 (71.4–71.8)	53.4 (52.9–53.8)	71.0 (70.7–71.2)	52.8 (51.0–52.6)	72.8 (72.5–73.1)	54.2 (53.7–54.7)
***Diet and beverage consumption***						
***Vegetables***						
Once a day or more	29.7 (29.5–29.9)	28.5 (28.1–28.9)	24.7 (24.5–25.0)	22.4 (21.7–23.1)	35.8 (35.4–36.1)	30.8 (30.3–31.3)
***Fruit***						
Once a day or more	27.2 (27.0–27.4)	24.8 (24.5–25.2)	21.5 (21.2–21.7)	17.8 (17.2–18.4)	34.2 (33.9–34.6)	27.3 (26.9–27.8)
***Whole grain bread***						
Once a day or more	32.5 (33.3–32.7)	25.7 (25.4–26.1)	32.6 (32.4–32.9)	27.5 (26.7–28.2)	32.3 (32.0–32.6)	25.1 (24.7–25.6)
***Fish***						
Once a week or more	79.0 (78.8–79.2)	71.1 (70.7–71.5)	79.8 (78.6–79.1)	72.7 (70.9–72.4)	79.3 (79.0–79.5)	70.8 (70.4–71.3)
***Salty snacks***						
< 4–6 times a week	93.9 (93.8–94.1)	89.3 (89.1–89.6)	93.3 (93.2–93.5)	86.6 (86.1–87.2)	94.7 (94.6–94.9)	90.3 (90.0–90.6)
***Candy***						
< 4–6 times a week	91.8 (91.7–91.9)	85.8 (85.5–86.1)	92.5 (92.3–92.7)	86.2 (85.6–86.7)	90.9 (90.7–91.1)	85.7 (85.3–86.0)
***Sugar-sweetened beverages***						
< 4–6 times a week	79.4 (79.2–79.6)	73.4 (73.0–73.8)	76.0 (75.8–76.3)	66.6 (65.8–67.4)	83.6 (83.3–83.8)	75.9 (75.5–76.3)
***Diet beverages***						
< 4–6 times a week	89.2 (89.1–89.3)	84.0 (83.7–84.4)	88.5 (88.3–88.7)	82.5 (81.8–83.1)	90.0 (89.8–90.2)	84.6 (84.3–85.0)
***Energy drinks***						
< 4–6 times a week	93.5 (93.4–93.6)	89.1 (88.9–89.4)	90.4 (90.2–90.6)	80.4 (79.8–81.1)	97.3 (97.2–97.5)	92.4 (92.1–92.6)
***Perceived family economy***						
Good economy	81.2 (81.0–81.4)	64.2 (63.8–64.7)	82.2 (81.9–82.4)	65.4 (64.7–66.2)	80.1 (79.8–80.4)	64.9 (63.4–64.4)
Nor bad or good	15.2 (15.1–15.4)	24.2 (23.9–24.6)	14.2 (14.0–14.4)	21.9 (21.2–22.6)	16.4 (16.1–16.6)	25.0 (24.6–25.4)
Bad economy	3.6 (3.5–3.7)	11.5 (11.3–11.8)	3.6 (3.5–3.7)	12.7 (12.1–13.2)	3.5 (3.4–3.7)	11.1 (10.8–11.4)
***Grade***						
8	21.4 (21.3–21.6)	11.5 (11.3–11.8)	20.7 (20.5–21.0)	11.4 (10.8–11.9)	22.5 (22.2–22.8)	11.7 (11.3–12.0)
9	19.3 (19.2–19.5)	16.5 (16.2–16.8)	19.4 (19.2–19.7)	15.5 (14.9–16.1)	19.3 (19.1–19.6)	16.9 (16.6–17.3)
10	18.1 (18.0–18.3)	19.8 (19.5–20.1)	18.4 (18.1–18.6)	19.6 (19.0–20.3)	17.7 (17.4–18.0)	19.8 (19.4–20.2)
11	18.3 (18.1–18.4)	21.5 (21.1–21.8)	18.8 (18.6–19.1)	22.4 (21.7–23.1)	17.4 (17.2–17.7)	20.1 (20.7–21.5)
12	14.3 (14.2–14.5)	17.7 (17.4–18.1)	14.8 (14.6–15.1)	18.8 (18.2–19.5)	13.7 (13.5–13.9)	17.4 (17.0–17.8)
13	8.5 (8.4–8.6)	13.0 (12.7–13.2)	7.8 (7.6–8.0)	12.3 (11.8–12.8)	9.3 (9.1–9.5)	13.2 (12.8–13.5)
***Parents higher education***						
Boths parents	61.5 (61.3–61.7)	53.7 (53.2–54.1)	60.6 (60.3–61.0)	53.6 (52.7–54.4)	62.6 (62.3–63.0)	53.7 (53.2–54.2)
One of the parents	21.4 (21.2–21.6)	24.9 (24.5–25.3)	21.7 (21.4–22.0)	24.4 (23.7–25.2)	21.0 (20.7–21.3)	25.1 (24.6–25.6)
None of the parents	17.1 (16.9–17.3)	21.5 (21.1–21.8)	17.7 (17.4–17.9)	22.0 (21.3–22.7)	16.4 (16.1–16.7)	21.2 (20.8–21.7)

High level of depressive symptoms coded as ≥80th percentiles and low level of depressive symptoms coded as <80th percentiles

A significantly higher proportion of girls reported a high level of depressive symptoms compared with boys (31.7% vs. 12.3%). Overall, and in gender subgroups, those with a high level of depressive symptoms reported a significantly poorer family economy compared with the rest of the study population.

Among students with a low level of depressive symptoms, a higher proportion participated in physical activity 3 or more times per week and spent 3 h or less per day on social media or gaming, compared with those with a high level of depressive symptoms. Furthermore, a higher proportion reported high consumption of fruit, whole-grain bread and low consumption of salty snacks, candy, sugar-sweetened beverages, diet beverages and energy drinks. Additionally, a higher proportion reported no alcohol-intoxicating episodes during the previous 12 months and no use of tobacco, including smokeless forms. These patterns were observed in boys and girls separately.

However, there were differences due to age: Among those with a high level of depressive symptoms, a higher proportion of students were in grades 10–13.

Results from a binary multivariable logistic regression with the dichotomised depressive symptoms scale as the dependent variable for girls and boys are provided in Table 3.

**Table 3:** Multivariable logistic regression of depressive symptoms among girls and boys in relation to lifestyle habits

	Girls	Boys
**Variables**	**AOR (95% CI)**	**AOR (95% CI)**
***Physical activity***		
< 3 times a week	1 (ref)	1 (ref)
≥ 3 times a week	0.83 (0.81–0.86)***	0.81 (0.78–0.85)***
**Social media use**		
> 3 times per day	1 (ref)	1 (ref)
≤ 3 times per day	0.70 (0.68–0.73)***	0.65 (0.62–0.68)***
**Gaming**		
> 3 times per day	1 (ref)	1 (ref)
≤ 3 times per day	0.77 (0.74–0.81)***	0.72 (0.69–0.75)***
**Smoking**		
Current use	1 (ref)	1 (ref)
No current use	0.72 (0.68–0.75)***	0.74 (0.69–0.79)***
**Smokeless tobacco**		
Current use	1 (ref)	1 (ref)
No current use	0.82 (0.78–0.86)***	0.94 (0.88–1.00)
**Alcohol intoxication (previous 12 months)**		
Any intoxication episodes	1 (ref)	1 (ref)
No intoxication episodes	0.67 (0.65–0.70)***	0.66 (0.62–0.70)***
**Vegetables**		
Less than every day	1 (ref)	1 (ref)
Every day and more	1.09 (1.05–1.14)***	1.11 (1.04–1.17)**
**Fruit**		
Less than every day	1 (ref)	1 (ref)
Every day and more	0.92 (0.88–0.96)***	0.94 (0.88–1.002)
**Whole grain bread**		
Less than every day	1 (ref)	1 (ref)
Every day and more	0.89 (0.85–0.92)***	0.91 (0.87–0.96)***
**Fish**		
Less than once a week	1 (ref)	1 (ref)
Once a week or more	0.80 (0.78–0.83)***	0.87 (0.82–0.91)***
**Salty snacks**		
≥ 4–6 times a week	1 (ref)	1 (ref)
< 4–6 times a week	0.86 (0.80–0.91)***	0.81 (0.74–0.88)***
**Candy**		
≥ 4–6 times a week	1 (ref)	1 (ref)
< 4–6 times a week	0.81 (0.77–0.85)***	0.77 (0.72–0.84)***
**Sugar-sweetened beverages**		
≥ 4–6 times a week	1 (ref)	1 (ref)
< 4–6 times a week	0.91 (0.87–0.94)***	0.99 (0.94–1.05)
**Diet beverages**		
≥ 4–6 times a week	1 (ref)	1 (ref)
< 4–6 times a week	0.84 (0.80–0.88)***	0.85 (0.80–0.90)***
**Energy drinks**		
≥ 4–6 times a week	1 (ref)	1 (ref)
< 4–6 times a week	0.62 (0.58–0.67)***	0.77 (0.72–0.82)***

*AOR* adjusted odds ratio for age, perceived family economy and parents’ higher education; 95% confidence intervals. *p < 0.1, ** *p* < 0.05, *** *p* < 0.001

The odds of experiencing a higher level of depressive symptoms were lower for girls and boys who reported being physically active 3 or more times per week, compared with those who did physical activity less than 3 times per week. Furthermore, the odds of having a higher level of depressive symptoms were lower for girls and boys who spent 3 h or less per day on social media or gaming, compared with those who spent more than 3 h per day on screen time.

The odds of having a higher level of depressive symptoms were lower for girls and boys who reported a higher consumption of fruit, whole-grain bread and fish, compared with those who reported a lower consumption of these healthier food choices, and for girls and boys who reported a lower consumption of salty snacks, diet beverages, energy drinks and candy, compared with those who reported a higher consumption of unhealthy food choices. Additionally, the odds of having a higher level of depressive symptoms were greater for girls and boys who reported a higher consumption of vegetables, compared with those who reported a lower consumption. Finally, the odds of having a higher level of depressive symptoms were lower for girls and boys who reported no current smoking activity or alcohol intoxication during the previous 12 months, and for girls who reported no current use of smokeless tobacco compared with the rest of the population.

Results concerning association of adherence to lifestyle habits and depressive symptoms among adolescents are provided in Table 4.

**Table 4:** Association between adherence to healthy lifestyle habits and depressive symptoms among adolescents in the Ungdata Survey

Degree of adherence to healthy lifestyle habits	Girls	Boys
	AOR (95% CIs)	AOR (95% CIs)
Lowest LSH	1	1
Low LSH	0.66 (0.63–0.69)	0.64 (0.60–0.68)
Middle LSH	0.62 (0.60–0.65)	0.57 (0.54–0.61)
High LSH	0.57 (0.55–0.60)	0.50 (0.47–0.53)
Highest LSH	0.52 (0.50–0.55)	0.41 (0.38–0.44)

*AOR* adjusted odds ratio for perceived family economy, parents’ higher education and age

*LSH* lifestyle habits. Lowest LSH; 1st quintile, low LSH; 2nd quintile, middle LSH; 3rd quintile, high LSH; 4th quintile, highest LSH; 5th quintile

The highest degree of adherence to healthy lifestyle habits was associated with reduced odds of depressive symptoms. Moving from less to more healthy lifestyle habit adherence implied lower odds of depressive symptoms.

## Discussion

This study contributes to the field by examining how depressive symptoms are related to a range of lifestyle habits, including physical activity, screen use, gaming, dietary habits, alcohol intoxication, smoking and smokeless tobacco. Overall, the present study’s main finding was that depressive symptoms were less common among adolescents with healthy lifestyle habits. Our results confirmed an inverse association between consumption of several healthy food items and prevalence of depressive symptoms and a positive association between consumption of several unhealthy food items and beverages, and prevalence of depressive symptoms. However, the results unexpectedly also indicated a weak positive association between consumption of vegetables and having depressive symptoms. In contrast to our findings, a meta-analysis concluded that fruit and vegetable consumption was associated with guarding against depression, thereby supporting the current recommendation of increasing fruit and vegetable consumption to improve mental health [41]. The weak positive association between consumption of vegetables and elevated depressive symptoms could be explained by the reverse causality effect, and the strong association with other possible confounders – such as social competence, family cohesion [42] and appearance satisfaction [5] – which were not included in the present study. Furthermore, recommendations on diet and soft drink consumption to prevent depression specifically have underlined the importance of high consumption of fruits, vegetables, legumes, whole-grain cereals, nuts, seeds and fish, and low consumption of processed foods, ‘fast’ foods, commercial baked goods, and sweets [13, 43]. These patterns also were confirmed in a recent meta-analysis of prospective studies, indicating that a higher-quality diet is associated with a lower risk of depressive symptoms [44]. However, other studies have not been able to identify any association between consumption of fish, fruit and vegetables, and depressive symptoms among adolescents [7, 45].

Furthermore, results from the present study indicated a strong positive association between consumption of energy drinks and depressive symptoms, which corresponds with results from a previously published systematic review [46]. Results from the present study also found a weak positive association between sugar-sweetened beverages and depressive symptoms among girls, but not boys. Previously published studies confirmed an association between soft drink consumption and depressive symptoms regardless of gender [47] and in females [48]. However, extant studies have not been able to identify any association between soft drinks and depressive symptoms [49], and between low-quality diets and higher depression incidence [44]. Inconsistent results from previous studies that have examined the relationship between diet and mental health in children and adolescents may be explained partly by the use of various measures of dietary factors, mental health problems and low methodological quality [10].

Findings from the present study also indicated an association between higher levels of physical activity and fewer depressive symptoms. This corresponds with a meta-analysis of prospective cohort studies indicating that higher levels of physical activity help guard against future development of depression [16], and a review of reviews found partial evidence for a causal association between physical activity and depression [50]. On the other hand, a study among UK adolescents did not reveal any association between physical activity and depressive symptoms [17]. Physical activity is a complex measure, and little is known regarding the mechanisms between physical activity and mental health [51]. How depressive symptoms and physical activity are operationalised and measured also may impact the relationship.

Both social media use and gaming were associated with a high prevalence of depressive symptoms in the present study, which corresponds with some [14, 18], but not all [52, 53], previous studies. A study among adolescents reported that social media use was associated with slight increases in depression, alcohol consumption and behavioural problems [54]. The increasing availability of screen-based devices has resulted in more time spent on social media and online gaming among adolescents, compared with previous generations [55], and may explain discrepancy patterns, and that type and features of online activities might affect mental health in different ways [56].

Corresponding with our results, previous studies also have confirmed an association between use of tobacco, alcohol intoxication and prevalence of depressive symptoms among adolescents [21, 22, 42]. Strandheim et al. [22] found that girls with symptoms of anxiety and depression reported more frequent alcohol intoxication, suggesting a bidirectional relationship. A systematic review concluded that internalising problems, including depression, was associated positively with more severe outcomes, such as heavy problematic drinking and alcohol use disorders, but associated negatively with alcohol consumption [57].

The present study revealed some minor gender differences in the associations between different lifestyle variables and depressive symptoms. Previous studies also have revealed gender differences; however, the impact from certain lifestyle factors varies. A previous study among Norwegian adolescents reported that alcohol and high levels of screen time were associated significantly with depressive symptoms among girls, while marijuana use, tobacco use and excessive screen time were associated significantly with depressive symptoms among boys [5]. Furthermore, among Australian adolescents, boys with unhealthy dietary patterns and lower physical activity levels were more likely to experience depressive symptoms, while higher levels of screen time were associated with depressive symptoms in girls [7]. Low appearance satisfaction has been revealed as an important confounder, particularly among females in the association between lifestyle risk and symptoms of depression [5]. This discrepancy could be explained by the broad range of lifestyle habits used in our study, the absence of confounding factors and that few previously published studies have reported on the association between multiple unhealthy and healthy lifestyle habits and depressive symptoms in adolescents.

The present study indicates differences between adolescents with lower SES compared with those who have higher SES. Adolescents who reported perceiving a good family economy had lower odds of experiencing depressive symptoms compared with the rest of the population. This corresponds with a review that found higher SES to be associated with fewer mental health problems among young people [30]. Additionally, depressive symptoms have been found to be more common among adolescent girls with lower SES compared with girls with higher SES [58]. However, results seem to vary across different SES indicators chosen in various studies. Lund et al. [58] suggested – based on a systematic review investigating the social gradient in stress and depressive symptoms among adolescent girls – that parental education, as an SES indicator, should be applied with care, although perceived financial situation seemed to be the most consistent SES measure across studies. A validity study comparing self-reported SES (parents’ occupations, Family Affluence scale and adolescents’ perception of their families’ socioeconomic, financial or social status) among adolescents suggested that although different indicators measure different SES dimensions, only perceived SES demonstrated a significant gradient in health-related quality of life [59].

We observed that higher, as opposed to lower, adherence to healthy lifestyle habits implied lower odds of having depressive symptoms, suggesting a need for multiple lifestyle changes. Similar to our results, previous studies have reported that mental health status was associated strongly with the number of health-promoting behaviours endorsed [4], and that increased numbers of risk behaviours were associated with an increased likelihood of depressive symptoms [5]. Additionally, adolescents meeting recommendations for multiple lifestyle factors had fewer physician visits for mental illness than those meeting recommendations for a single behaviour [8].

The association of diet and beverage consumption and depression may be explained by inflammatory pathways [60]. Adopting an anti-inflammatory diet and diets that include omega-3-polyunsaturated fatty acids and dietary fibre might be linked to reduced risk of developing symptoms of depression [61]. Although biological arguments have been made for a possible causal direction between diet and depression, reverse caution must be addressed, as it is plausible that the relationship could be bi-directional, and that depression could impact lifestyle habits. For example, emotions may influence eating behaviour both in terms of the choice of nutrition and volume consumed [62, 63]. Furthermore, depressive symptoms and depression also have been found to affect physical activity [64] and increase risks from sedentary behaviour [65].

Lifestyle habits in previous studies have been shown to cluster or coexist with one another and have been associated with different levels of mental health symptoms [6, 66]. Several possible mechanisms may contribute to this. As demonstrated above, depressive symptoms, such as anhedonia and withdrawal, may make individuals predisposed to having more negative lifestyle habits, and Verger et al. [67] suggested that diminished interest in health and being less receptive to health education messages could be one explanation for the clustering of negative lifestyle habits among those with depression. Furthermore, common background factors such as SES may influence both various lifestyle factors and depressive symptoms.

Examining lifestyle habits that can explore underlying mechanisms and improve mental health in both clinical and public health settings is vital to future research. Youth counsellors also should be aware of the relationship between depressive symptoms and lifestyle habits. However, although there are potential benefits at the individual level from engaging in healthy lifestyle habits, the present findings are also important for guiding health policies. The positive effects from adopting a healthy lifestyle, present opportunities for implementing health promotion interventions in schools, e.g., as part of a cross-sectoral strategy for public health. Public health strategies should include measures that facilitate the possibility of healthy lifestyle choices for all adolescents, e.g., through universal interventions such as school meals, safe environments that promote physical activity and reduced pricing for healthy food products.

### Strengths and limitations

The present study’s major strengths were the large sample size combined with a high response rate. The data used in this study were collected in 2017–2019 and provide an up-to-date description of Norwegian adolescents’ lives. The outcome measure worked well psychometrically on a general level, but one item, ‘worried too much about things’, worked differently for boys and girls, and another item, ‘had sleep problems’, clearly misfits [36]. A limitation in the present study was the cross-sectional design, which precludes inferences about causal relationships. Depressive symptoms may function not only as an outcome, but also may act as an exposure that is hampering lifestyle factors and can influence how young people evaluate their lifestyle and family economy. Furthermore, the use of self-reported measures may have led to unidentified misclassification or measurement errors. As far as we know, no evaluation has been performed in the Ungdata Survey regarding self-reporting of physical activity, screen time or diet and beverage intake. Furthermore, the associations found between different lifestyle habits and depressive symptoms might be influenced by other factors that were not controlled for in the present study. Thus, the results should be interpreted with caution.

## Conclusion

Results from the present study indicate that healthier lifestyle habits were associated with a lower level of depressive symptoms in adolescents, as well as demonstrate support for the interrelationship between lifestyle habits and depressive symptoms. Adolescence is a critical time for establishing healthy lifestyle habits, and we hope that our results can contribute to a better understanding of supporting investment in adolescents’ adherence to a healthier lifestyle and be useful for general practice in promoting adolescents’ mental health. Moreover, the findings confirmed the positive consequences of practising healthy lifestyles during childhood and adolescence. Further research is needed to examine the direction and causality between these associations.

## Data Availability

The data and materials from the Ungdata Surveys are closed and stored in a national database administered by NOVA. The data are available for research purposes upon application. For request of the data, please contact ungdata@oslomet.no. Further information about the study and the questionnaires can be found on the web page (in Norwegian) (http://ungdata.no/).

## Abbreviations

OR: Odds ratio
NOVA: Norwegian Social Research
KoRus: Regional Drug and Alcohol Competence Centres
LSH: Lifestyle habits
CI: Confidence interval
SES: Socioeconomic status
